# Continuity up to the boundary for minimizers of the one-phase Bernoulli problem

**DOI:** 10.1007/s00526-025-03040-3

**Published:** 2025-05-27

**Authors:** Xavier Fernández-Real, Florian Gruen

**Affiliations:** 1 Institute of Mathematics, École Polytechnique Fédérale de Lausanne, Lausanne, Switzerland; 2 Department of Mathematics, Graduate School of Science, Kyoto University, Kyoto, Japan

**Keywords:** 35R35, 35B65, 35N25, 35A02

## Abstract

We prove new boundary regularity results for minimizers to the one-phase Alt-Caffarelli functional (also known as Bernoulli free boundary problem) in the case of continuous and Hölder-continuous boundary data. As an application, we use them to extend recent generic uniqueness and regularity results to families of continuous functions.

## Introduction

In this work, we study minimizers of the *Alt–Caffarelli functional*

1.1 $$\begin{aligned} {{\,\mathrm{\textit{F}_\Lambda }\,}}(u,D) :=\int _{D} |\nabla u|^2 dx + \Lambda |\{u>0\} \cap D|, \end{aligned}$$

where *D* is an open domain in ${{\,\mathrm{\mathbb {R}}\,}}^d$, $\Lambda$ a positive real constant and $u\in H^1(D)$.

The problem, also known as one-phase or Bernoulli problem, originates in the fundamental works [1, 4] and has various applications in models of flame propagation [3] and jet flows [2]. Recent developments include [6, 7, 9–11, 13, 15]. We also refer to [5] and [17] for a detailed mathematical exposition.

Given an open bounded domain $D\subset {{\,\mathrm{\mathbb {R}}\,}}^d$ and a boundary datum $g\in H^1(D)$ with $g\ge 0$ in *D*, the Alt–Caffarelli problem is the minimization:

1.2 $$\begin{aligned} \min \{ {{\,\mathrm{\textit{F}_\Lambda }\,}}(u,D): u\in H^1(D) \text { such that } u-g \in H_0^1(D)\}. \end{aligned}$$

Any minimizer *u* is nonnegative, and splits the domain into two parts:

$$ \Omega _u:=\{x \in D :u(x)>0\}\quad \text { and } \quad \Omega _0:=\{x \in D :u(x)=0\}. $$

The interface between the two sets, $\partial \Omega _u$, is a priori unknown and is called the *free boundary*.

Any minimizer *u* to (1.2) is locally Lipschitz continuous inside *D* (see e.g. [17, Chapter 3]). The Euler–Lagrange equation, satisfied by (classical) stationary points of (1.1), is given by

$$ \left\{ \begin{array}{rcll} u &  \ge &  0 &  \quad \text {in}\quad D,\\ \Delta u &  = &  0 &  \quad \text {in}\quad \Omega _u,\\ |\nabla u | &  = &  \sqrt{\Lambda }&  \quad \text {on}\quad \partial \Omega _u\cap D. \end{array} \right. $$

For general stationary solutions, the previous equations need to be understood in the viscosity sense. Throughout the paper, for the sake of simplicity, we will fix $\Lambda = 1$.

Reminiscent of the classical Laplace equation with Dirichlet boundary condition^1^, the main goal of this work is to establish basic regularity estimates up to the boundary for solutions to (1.2). To our knowledge, up until now, the community has been proving such estimates on a need-to-use basis (see [9, 13]). We hope that this short note can be useful to researchers in contexts where such estimates can be applied. In this direction, we present some examples of applications of our results, namely a comparison principle and generic-type results for minimizers.

### Main results

Our main result says that minimizers of the one-phase problem with continuous boundary datum are continuous up to the boundary. The following result applies, for example, to the case of $C^1$ domains.

#### Theorem 1.1

Let $d\ge 2$ and $D \subset {{\,\mathrm{\mathbb {R}}\,}}^d$ be an open domain such that

- either *D* is convex,
- or *D* is a locally *c*-Lipschitz domain, for some *c* small enough depending only on *d*.

Let $g\in C(\overline{D}) \cap H^1(D)$ with modulus of continuity $\omega$ and $\Vert g\Vert _{H^1(D)}\le M$ for some $M > 0$, and let *u* be a minimizer to (1.2).

Then, $u \in C(\overline{D})$, with a modulus of continuity depending only on $\omega$, *D*, $\Lambda$, and *M*. That is, for any $\varepsilon >0$, there exists $\delta =\delta (\omega , D, \Lambda , M)$ such that

$$\begin{aligned} |x-y|< \delta \implies |u(x)-u(y)| < \varepsilon \qquad \forall x,y \in \overline{D}. \end{aligned}$$

A priori, as for the case of harmonic functions, the modulus of continuity of *u* does not need to be the same (nor comparable) to the modulus of continuity of *g*.

This is in contrast to the case of more regular boundary data, where for Hölder coefficients we actually obtain Hölder regularity up to the boundary (again, as for harmonic functions):

#### Proposition 1.2

Let $d\ge 2$ and $D \subset {{\,\mathrm{\mathbb {R}}\,}}^d$ be an open bounded $C^{1,\alpha }$ domain. Let $g\in C^\gamma (\bar{D}) \cap H^1(D)$ where $\frac{1}{2}<\gamma <1$, and let *u* be a minimizer to (1.2). Then, $u \in C^\gamma (\overline{D})$ and

$$\begin{aligned} \Vert u\Vert _{C^{\gamma }( \overline{D})} \le C \left( 1+ \Vert g\Vert _{C^{\gamma }(\partial D )}+ \Vert u\Vert _{L^\infty ( D)} \right) , \end{aligned}$$

where *C* depends only on *d*, $\gamma$, $\Lambda$, $\alpha$ and *D* (in particular, through its diameter and $C^{1,\alpha }$ norm; see Definition 2.2).

This result is a generalization of the case for $\gamma = 1$, originally treated in [9, Appendix B]; the condition $\gamma >\frac{1}{2}$ is sufficient to ensure that the boundary datum *g* lies in $H^1(D)$, see also Remark 2.8.

### Applications to generic regularity

In the second part of the paper, we apply the continuity up to the boundary to show how to extend the results on generic uniqueness of minimizers from [13] to the case of merely continuous data.

Namely, we show:

#### Proposition 1.3

Let $d \ge 2$, and let $D\subset {{\,\mathrm{\mathbb {R}}\,}}^d$ be a domain as in Theorem 1.1. Let $g_t\in C(\overline{D})\cap H^1(D)$ for $t\in (0, 1)$ with $\sup _{t\in (0, 1)}\Vert g_t\Vert _{H^1(D)}< \infty$ be such that, for all $0< s< t< 1$,

(i) $$g_t\ge g_s\ge 0$$ in *D*, and
(ii) any connected component of $\{g_s > 0\}\cap \partial D$ contains $x_0$ with $g_t(x_0) > g_s(x_0)$.

Then, there exists a countable subset $J \subset (0,1)$ such that for every $t \in (0,1) \backslash J$, there is a unique minimizer of $F_\Lambda (\cdot , D)$ with boundary datum given by $g_t$.

Lastly, we also show a generic regularity result for the free boundary. By [18], it is already known that up to a certain critical dimension $d^*$ ($4\le d^* \le 6$, see [8, 15]) the free boundary of *u* is always smooth, i.e. its set of singular points, denoted $\textrm{Sing}(u)$, is empty (and in general dimension, it has Hausdorff dimension $d-d^*-1$). Thanks to [13], generically this dimension can be increased by one if one takes minimizers with Lipschitz boundary data. We generalize the result to a wider class of boundary data:

#### Theorem 1.4

Let $d \ge 2$, and let $D\subset {{\,\mathrm{\mathbb {R}}\,}}^d$ be a domain as in Theorem 1.1. Let $g_t\in C(\overline{D})\cap H^1(D)$ with $g_t \ge 0$ for $t\in (0, 1)$, $\sup _{t\in (0, 1)}\Vert g_t\Vert _{H^1(D)} <\infty$, and

$$ g_t - g_s \ge t-s\quad \text {in}\quad D \quad \text {for all}\quad 0< s< t< 1. $$

Let $u_t$ denote any minimizer of $F_\Lambda (\cdot , D)$ with boundary datum $g_t$. Then:

- If $d=d^*+1$, there exists a countable subset $J \subset (0,1)$ such that
  $$\begin{aligned} \textrm{Sing}(u_t) = \varnothing \qquad \qquad \text {for every } t \in (0,1) \backslash J. \end{aligned}$$
- If $$d\ge d^*+2$$,
  $$\begin{aligned} \text {dim}_{{{\,\mathrm{\mathcal {H}}\,}}} \, \textrm{Sing}(u_t) \le d- d^*-2 \quad \quad \text {for almost every } t \in (0,1), \end{aligned}$$

where $\textrm{dim}_{\mathcal {H}}$ denotes the Hausdorff dimension of a set.

#### Remark 1.5

As an example, the family $\{g+\lambda \}_{\lambda \in (0,1)}$ with $g:\partial D \rightarrow {{\,\mathrm{\mathbb {R}}\,}}$ nonnegative and continuous, satisfies the assumptions of Proposition 1.3 and Theorem1.4.

#### Remark 1.6

Contrary to [13], where the family ${g_t}$ is required to be equi-Lipschitz continuous, any assumption on equicontinuity becomes redundant and only uniform boundedness of the family ${g_t}$ and monotonicity are required.

We finally also refer to Lemma 3.1 for a comparison principle between minimizers with continuous boundary data.

### Structure of the paper

We start by proving, in Subsection 2.1 and by means of a barrier and compactness argument, the quantitative continuity up to the boundary, Theorem 1.1. In Subsection 2.2, we then show Proposition 1.2: for Hölder continuous boundary datum, the minimizer is also Hölder continuous (with the same exponent) up to the boundary. For that, we use a modified version of the arguments in [9, Lemma B.1].

Finally, in Section 3, we apply our results by first proving a general comparison lemma for continuous minimizers, and then to show generic uniqueness (Proposition 1.3 in Subsection 3.1) and generic regularity (Theorem 1.4 in Subsection 3.2). There we show how to modify the arguments from [13], and how to work around the equicontinuity of the boundary data.

## Boundary regularity

This section introduces the two new boundary regularity results. Note that the regularity of the ambient domain is crucial as well, however here we are not concerned with necessary conditions (*Wiener-type criteria*) and assume sufficient regularity of $\partial D$ as needed. We recall that the hypograph of a function $f:{{\,\mathrm{\mathbb {R}}\,}}^d \rightarrow {{\,\mathrm{\mathbb {R}}\,}}$ is given as

$$\begin{aligned} {{\,\textrm{hyp}\,}}(f) := \{(x,y) \in {{\,\mathrm{\mathbb {R}}\,}}^{d+1}: f(x) \ge y \}. \end{aligned}$$

We also state some standard definitions here for the reader’s convenience.

### Definition 2.1

A domain $D \subset {{\,\mathrm{\mathbb {R}}\,}}^d$ is *c*-Lipschitz for some $c > 0$, if for each $x_0\in \partial D$, up to a rotation, $\partial D$ is the graph of a *c*-Lipschitz function $\varphi$ in $B_1(x_0)$.

### Definition 2.2

A domain $D\subset {{\,\mathrm{\mathbb {R}}\,}}^d$ is a $C^{1,\alpha }$ domain for some $\alpha \in (0, 1]$, if for each $x_0\in \partial D$, up to a rotation, $\partial D$ is the graph of a $C^{1,\alpha }$ function $\varphi$ in $B_1(x_0)$. The maximum $C^{1, \alpha }$ norm of such function among all $x_0\in \partial D$ is what we call the $C^{1,\alpha }$ norm of the domain *D*.

### Remark 2.3

Up to a rescaling, any bounded domain that is locally Lipschitz/$C^{1,\alpha }$ is a Lipschitz/$C^{1,\alpha }$ domain respectively.

### Continuous boundary datum

The first result we prove concerns the regularity of minimizers with merely continuous datum. We recall the well-known solution on an annulus. Remember that we are fixing $\Lambda = 1$.

#### Proposition 2.4

[17, Proposition 2.15] Let $d\ge 2$. There exists $R=R(d)\in (1, 2)$ such that

$$\begin{aligned} v_1(x) := {\left\{ \begin{array}{ll} 1-\frac{\log |x|}{\log R} & \text { if }d=2,\\[7pt] \frac{|x|^{2-d} - R^{2-d}}{1 - R^{2-d}} & \text { if }d\ge 3,\\ \end{array}\right. } \end{aligned}$$

is the unique solution of $(1.2)$ on $A= B_R\backslash B_1$ with $g|_{\partial B_1}=1$ and $g|_{\partial B_R}=0$.

Using the previous explicit solution as a barrier, we are able to prove quantitatively that the minimizer *u* is continuous up to the boundary (Theorem 1.1). The modulus of continuity of the solution *u* is not necessarily the same as for the boundary datum, but depends on it (as well as the domain and the modulus of the boundary datum itself). We start with a lemma, stating that minimizers are positive close to positive boundary data:

#### Lemma 2.5

Let $d\ge 2$, and let *D* be an open domain such that

- either *D* is convex,
- or *D* is *c*-Lipschitz, for some *c* small enough depending only on *d*.

Let us assume, moreover, that $0\in \partial D$, and that $u \ge 2 > 0$ on $\partial D\cap B_2$. Then,

$$ u > 0 \quad \text {in}\quad D\cap B_\rho , $$

for some $\rho > 0$ depending only on *d*.

#### Proof

**Fig. 1:**
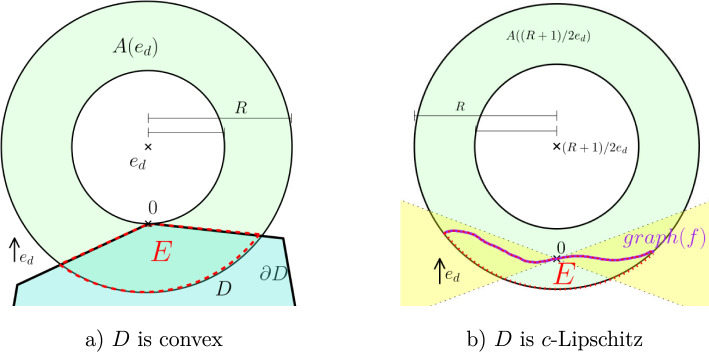
The set-up for the proof of Theorem 1.1

We proceed with a barrier argument (see Figure 1 for a sketch of the setting in the two types of domain).

Take the annulus *A* from Proposition 2.4. We now consider the two cases:

- *D* **is convex:** Up to a rigid motion, we assume that $D \subset H$, where $H = \{(x',x_d): x_d\le 0\}$. Set
  $$ E:= A(e_d) \cap D, \qquad \partial E = E_1 \cup E_2 $$
  where
  $$ E_1 := \partial D \cap A(e_d) \subset \partial D \cap B_1, \qquad E_2 :=\partial B_{R} (e_d) \cap D. $$
  Let $$v_1$$ be the (unique) solution from Proposition 2.4 in the annulus *A*. Then for $$ v(x):= v_1(x+ e_d)$$, on $$E_1$$,
  $$ v(x)\le 1 <2 \le u(x)\quad \text {on}\quad E_1, $$
  and $$ v(x) =0$$ on $$E_2$$. In particular, we get that
  $$ v\le u\quad \text {on}\quad \partial E. $$
- $\partial D$ **is** *c***-Lipschitz:** Without loss of generality (up to rotation and rescaling), we assume that *D* is the subgraph/hypograph of a *c*-Lipschitz function $f:B^{d-1}_2 \rightarrow {{\,\mathrm{\mathbb {R}}\,}}$ in the $e_d$ direction and $f(0)=0$ (denote here by $B_2^{d-1}$ the $(d-1)$-dimensional ball of radius 2). Since *f* is *c*-Lipschitz, we have that for any $0<h<1$ the graph of *f* lies within $B_h^{d-1}\times [-ch,ch]$. Then (recall $R\in (1, 2)$ is given by Proposition 2.4, so $\frac{R+1}{2}\in (1, R)$), we have
  $$ {{\,\textrm{graph}\,}}(f) \cap A\left( \frac{R+1}{2} e_d \right) \subset B_{2}^{d-1} \times [-2c, 2c]. $$
  Set now *E* to be the connected component of
  $${{\,\textrm{hyp}\,}}(f) \cap A\left( \frac{R+1}{2}e_d \right) $$
  containing the origin. As long as $c\le \frac{R-1}{2}$, $\partial E = E_1 \cup E_2$ with
  $$E_1 \subset {{\,\textrm{graph}\,}}(f)\quad \text { and }\quad E_2 \subset \partial B_{R}\left( \frac{R+1}{2} e_d \right) . $$
  Let $v_1$ be the solution on the annulus *A*, for
  $$v(x):= v_1 \left( x+\frac{(R+1)}{2}e_d \right) $$
  we have
  $$ v(x)\le 1 <2 \le u(x) \qquad \text { on } E_1 $$
  and $v(x)=0$ on $E_2$. In particular, we get again that
  $$ v\le u \quad \text {on}\quad \partial E. $$

In both cases, we have constructed a set *E* where the boundary consists of two parts $E_1$ and $E_2$, and $u|_{E_1} > v|_{E_1}$ and $u|_{E_2}\ge v|_{E_2}=0$. Also $v|_E$ and $u|_E$ are minimizers on *E* for their own boundary datum, i.e.

$$ {{\,\mathrm{\textit{F}_\Lambda }\,}}(v,E) \le {{\,\mathrm{\textit{F}_\Lambda }\,}}(\min (v,u), E) \qquad \text {and} \qquad {{\,\mathrm{\textit{F}_\Lambda }\,}}(u,E) \le {{\,\mathrm{\textit{F}_\Lambda }\,}}(\max (v,u), E). $$

From the cut-and-paste lemma for the one-phase problem (see [17, Lemma 2.5]),

$$\begin{aligned} {{\,\mathrm{\textit{F}_\Lambda }\,}}(\min (v,u),E) + {{\,\mathrm{\textit{F}_\Lambda }\,}}(\max (v,u),E) = {{\,\mathrm{\textit{F}_\Lambda }\,}}(v,E) + {{\,\mathrm{\textit{F}_\Lambda }\,}}(u,E), \end{aligned}$$

thus ${{\,\mathrm{\textit{F}_\Lambda }\,}}(v,E)= {{\,\mathrm{\textit{F}_\Lambda }\,}}(\min (v,u),E)$. Since *v* as a minimizer is unique, we have $v=\min (v,u)$, i.e. $u\ge v$ with *v* vanishing only on a subset of $\partial E$. Hence, there exists some small $\rho >0$ such that on $B_\rho \cap D$ the function *u* is strictly positive as was to be shown. $\square$

As a consequence we obtain the proof of the regularity up to the boundary:

#### Proof of Theorem 1.1

Assume by contradiction that it is not true. Then there exists $\bar{\varepsilon }>0$ and a sequence $\{g_k\}_{k\in \mathbb {N}}$ with $g_k \in C(\overline{D}) \cap H^1(D)$ having a uniform modulus of continuity $\omega$ and $\Vert g_k\Vert _{H^1(D)} \le M<\infty$ such that for some minimizers $u_k$ to (1.2) with $u_k-g_k=H_0^1(D)$, there exist $x_k,y_k \in \overline{D}$ such that

2.1 $$\begin{aligned} |x_k-y_k| \le \frac{1}{k}\rightarrow 0 \qquad \text {but} \qquad |u_k(x_k)-u_k(y_k)| \ge \bar{\varepsilon }>0,\qquad \text {for}\quad k\in \mathbb {N}. \end{aligned}$$

By the uniformity of the modulus of continuity $\omega$ and the boundedness of the $H^1$-norm of $g_k$, again up to a subsequence $g_k \rightarrow g_\infty$ for some $g_\infty$ with the same modulus of continuity $\omega$ and $\Vert g_\infty \Vert _{H^1(D)}\le M$. Note also that $u_k \rightarrow u_\infty$ in $H^1_{loc}(D)$ with $u_\infty$ being a minimizer of (1.2) with boundary datum $g_\infty$ by [17, Lemma 6.3]. Both, $u_k$ and $u_\infty$ are locally Lipschitz continuous in *D* independently of *k*.

Moreover, by compactness of *D*, $x_k$ and $y_k$ converge, up to a subsequence, to $x_0 \in \overline{D}$. We now separate between three cases:

**Case** $x_0 \in D$**:** For sufficiently large *k*, $x_k$, $y_k$, and $x_0$ are inside some $D'\subset \subset D$. By the interior uniform (Lipschitz) continuity of $u_k$ and $u_\infty$

we get a contradiction with (2.1).

**Case** $x_0 \in \partial D \cap \{g_\infty =0\}$**:** Since $g_k\rightarrow g_\infty$, $g_k(x_0) \rightarrow 0$ as well. Consider now the solution to the Dirichlet problem,

$$\begin{aligned} \left\{ \begin{array}{rlll} \Delta \bar{u}_k & =& 0 \qquad & \text {in }D\\ \bar{u}_k & =&  g_k \qquad & \text {on }\partial D. \end{array} \right. \end{aligned}$$

By the classical theory ([14, Lemma 2.13]), the $\bar{u}_k$’s have the same modulus of continuity $\bar{\omega }$, depending only on $\omega$, *d*, *D* and *M*. Thus

$$\begin{aligned} | \bar{u}_k(x_k)| \le |\bar{u}_k(x_k)-\bar{u}_k(x_0)| + |g_k(x_0)| \le \bar{\omega }(|x_k-x_0|) + |g_k(x_0)|, \end{aligned}$$

which vanishes as $k \rightarrow \infty$. Hence $\bar{u}_k(x_k), \bar{u}_k(y_k)\rightarrow 0$. On the other hand, from the subharmonicity of $u_k$ , by the comparison principle for weak (sub)solutions we have

$$\begin{aligned} |u_k(x_k) - u_k(y_k)| \le u_k(x_k) + u_k(y_k) \le \bar{u}_k(x_k) + \bar{u}_k(y_k) \rightarrow 0, \end{aligned}$$

again a contradiction with (2.1).

**Case** $x_0 \in \partial D \cap \{g_\infty >0\}$**:** We proceed by using the barrier argument from Lemma 2.5. Without loss of generality, up to a translation, we assume $x_0=0$ and observe that for some $\rho >0$ and for any *k* sufficiently large, $u_k>0$ in $B_\rho \cap D$, and therefore, the $u_k$’s are harmonic there.

Indeed, for *k* sufficiently large, and up to a rescaling by *r*, $\frac{u_k(r x)}{r}$ (independent of *k*), we can assume that $\frac{g_k}{r} \ge \frac{g_\infty (0)}{2 r}$ in $B_2 \cap \partial D$, so that, up to taking *r* smaller if necessary (such that $\frac{g_\infty (0)}{2 r}\ge 2$), we are in the setting of Lemma 2.5. Thus, there is a small $\rho > 0$ (independent of *k*) such that $u_k > 0$ in $D\cap B_\rho$, and thereby the $u_k$’s are harmonic there. We apply [14, Lemma 2.13] to get $u_k$ continuous in $\overline{B_{\rho /2} \cap D}$ with a common modulus of continuity $\bar{\omega }$, depending only on $\omega$, *d*, $\rho$, and *D*.

Thus

$$|u_k(x_k)-u_k(x_0)| \le \bar{\omega }(|x_k-x_0|), \qquad |u_k(y_k)-u_k(x_0)|\le \bar{\omega }(|y_k-x_0|)$$

and therefore, as $k \rightarrow \infty$, by the triangle inequality

$$\begin{aligned} |u_k(x_k)-u_k(y_k)| \le \bar{\omega }(|x_k-x_0|) + \bar{\omega }(|y_k-x_0|) \rightarrow 0, \end{aligned}$$

again a contradiction to (2.1). This finishes the proof. $\square$

### Hölder continuous boundary datum

For the case with Hölder continuous boundary datum, we show that the Hölder regularity is preserved. We do so by following [9]. First we state a well-known technical tool, the Morrey Lemma [17, Lemma 3.12].

#### Lemma 2.6

Let $\Omega \subset {{\,\mathrm{\mathbb {R}}\,}}^d$, $u\in H^1(\Omega )$ and for $C>0$, $\gamma \in (0,1)$Then $u \in C^{0,\gamma }(B_{R/8})$ with

$$\Vert u\Vert _{C^{0,\gamma }(B_{R/8})} \le C \left( 2^d + \frac{2}{\gamma } \right) . $$

We now can prove the local version of Proposition 1.2:

#### Proposition 2.7

Let *D* be an open bounded $C^{1,\alpha }$ domain in $B_1$, given by the subgraph of a function with $C^{1,\alpha }$ norm bounded by 1. Let *u* be a minimizer of (1.2) on *D* with boundary datum $g \in C^{\gamma }(\bar{D})\cap H^1(D)$ with $1>\gamma >\frac{1}{2}$, and $0\in \partial \{u > 0\}$.

Then,

$$\begin{aligned} \Vert u\Vert _{C^{\gamma }(B_{1/2} \cap \overline{D})} \le C \left( \Vert u\Vert _{C^{\gamma }( B_1 \cap \partial D )}+ \Vert u\Vert _{L^\infty (B_1 \cap D)} +1\right) , \end{aligned}$$

for some constant *C* depending only on *d*, $\gamma$, and $\alpha$.

#### Proof

Let $\partial D$ be the graph of a $C^{1,\alpha }$ function $\phi$ in $B_1$, i.e. $\partial D \cap B_1 = \{(x',\phi (x')): x' \in {{\,\mathrm{\mathbb {R}}\,}}^{d-1}\}$. It suffices to show $\gamma$-Hölder continuity in a small ball $B_{1/8}$. Denote the positive and negative half spaces by $H^+$ and $H^-$.

First, we extend $\nabla u:B_1 \cap D \rightarrow {{\,\mathrm{\mathbb {R}}\,}}^d$ to $\nabla u:B_1 \rightarrow {{\,\mathrm{\mathbb {R}}\,}}^d$. In order to do so, let $\Phi :B_1 \rightarrow {{\,\mathrm{\mathbb {R}}\,}}^d$ be the $C^{1,\alpha }$ function

$$\begin{aligned} \Phi (x',x_d) := (x', x_d + g(x')). \end{aligned}$$

Up to translation and rotation, we assume $\Phi (0)=0$ and $D\Phi (0)=I_{d\times d}$. Let

$$\pi :(y',y_d) \mapsto (y',-y_d),$$

we define the extension

$$\begin{aligned} \nabla u (x) = {\left\{ \begin{array}{ll} \nabla u (x) \qquad & \text {if } x \in D \cap B_1,\\ \nabla u ( \Phi \circ \pi \circ \Phi ^{-1}( x)) \qquad & \text {if } x \in D^c \cap B_1. \end{array}\right. } \end{aligned}$$

The idea is to arrive at an estimate for *u* of the form

2.2 $$\begin{aligned} \forall \bar{x} \in B_{1/8}, \forall r\le \frac{1}{2}: \qquad \int _{B_r(\bar{x})} |\nabla u|^2 \le Cr^{d+2(\gamma -1)}, \end{aligned}$$

and then use the Morrey Lemma [17, Lemma 3.12], which gives directly $\gamma$-Hölder regularity in $B_{1/8}$. As in [9], it suffices to show the estimate on the boundary, i.e. for a fixed $r>0$ small enough and $x_0 \in \partial D \cap B_{1/8}$,

2.3 $$\begin{aligned} \int _{B_r(x_0)} |\nabla u|^2 \le Cr^{d+2(\gamma -1)}. \end{aligned}$$

By a translation, we assume that $x_0=0$. Performing the change of variable,

$$x=\Phi (y),\qquad dx=|D\Phi (y)|dy,$$

we have

$$\begin{aligned} \begin{aligned}&\int _{B_r} |\nabla u(x)|^2 dx =\int _{\Phi (B_r)} |\nabla u(x)|^2 dx = \int _{B_r} |\nabla u(\Phi (y))|^2 |D\Phi (y)| dy\\&\qquad = \int _{B_r \cap H^+} |\nabla u(\Phi (y))|^2 |D\Phi (y)| dy + \int _{B_r \cap H^-} |\nabla u(\Phi \circ \pi \circ \Phi ^{-1}( \Phi (y)))|^2 |D\Phi (y)| dy \\&\qquad = 2\int _{B_r \cap H^+} |\nabla u(\Phi (y))|^2 |D\Phi (y)| dy = 2 \int _{\Phi (H^+ \cap B_r)} |\nabla u(x)|^2 dx. \end{aligned} \end{aligned}$$

**Step 1: **Let $h_g:H^+ \cap B_1 \rightarrow {{\,\mathrm{\mathbb {R}}\,}}$ such that

$$\begin{aligned} \left\{ \begin{array}{rlll} \Delta h_g & = & 0 \qquad & \text {in } H^+ \cap B_1, \\ h_g & = & g \circ \Phi \qquad & \text { on } \partial (H^+ \cap B_1), \end{array} \right. \end{aligned}$$

which is in $C^{\gamma }(\bar{H}^+ \cap B_{1/2})$ by [16, Proposition 2.1] (*u* is continuous in $\overline{D}$ by Theorem 1.1) with

$$\begin{aligned} \begin{aligned} \Vert h_g\Vert _{C^{\gamma }(H^+\cap B_{1/2})}&\le C_{d,\gamma } \left( \Vert g \circ \Phi \Vert _{C^{\gamma }(\partial H^+ \cap B_1)} + \Vert h_g\Vert _{L^\infty ( H^+ \cap B_1)} \right) \\&\le C \left( \Vert g \Vert _{C^{\gamma }(\partial D)} + \Vert g\Vert _{L^\infty (H^+ \cap B_1)} \right) , \end{aligned} \end{aligned}$$

for *C* depending on $d,\gamma$ and the $C^{1,\alpha }$ norm of *D*. We now claim that inside $H^+ \cap B_{1/2}$

$$ |\nabla h_g(x)|\le C_{d} \Vert h_g\Vert _{C^{\gamma }(H^+\cap B_{1/2})} {{\,\textrm{dist}\,}}(x,\partial H^+)^{\gamma -1}. $$

For a fixed *x*, Take $x_1\in \partial H^+$ such that $|x-x_1|={{\,\textrm{dist}\,}}(x,\partial H^+) =: \delta (x)$ and let

$$ v(x):=h_g(x)-h_g(x_1). $$

By $\gamma$-Hölder regularity of *v*, from the definition, for $x \in H^+ \cap B_{1/2}$

$$\begin{aligned} v(x) = v(x_1) + R(x)=R(x) \qquad \text {with } |R(x)|\le 2 \Vert h_g\Vert _{C^{\gamma }(H^+\cap B_{1/2})}|x-x_1|^{\gamma }. \end{aligned}$$

By standard harmonic estimates,

$$\begin{aligned} \begin{aligned} |\nabla h_g(x)|= |\nabla v(x)|&\le \sup _{B_{\delta (x)/4}(x)}|\nabla v(y)| \\&\le \frac{d}{\delta (x)} \sup _{B_{\delta (x)/2}(x)} |v(y)| \\&\le \frac{d}{\delta (x)} \sup _{B_{\delta (x)/2}(x)} |R(y)| \\&\le \frac{2 d\Vert h_g\Vert _{C^{\gamma }(H^+\cap B_{1/2})}}{\delta (x)} \sup _{B_{\delta (x)/2}(x)}|y-x_1|^{\gamma } \\  &\le 4 d \Vert h_g\Vert _{C^{\gamma }(H^+\cap B_{1/2})} \delta (x)^{\gamma -1}, \end{aligned} \end{aligned}$$

proving the claim. In the co-area formula [12, Theorem 3.2.22], take $f(x)= \delta (x)$ and $g(x) = \delta (x)^{2(\alpha -1)}$. Since $\partial H^+ =\{x_d=0\}$ we have $|\nabla f(x)|=1$. Hence, as

$$f^{-1}(t)=\{ x \in H^+\cap B_r: \delta (x)=t\},$$

we estimate for $r< \frac{1}{2}$,

$$\begin{aligned} \begin{aligned} \int _{H^+\cap B_r}|\nabla h_g|^2dx&\le C_{d} \Vert h_g\Vert ^2_{C^{\gamma }(H^+\cap B_{1/2})} \int _{H^+\cap B_r} \delta (x)^{2(\gamma -1)}dx\\&=C_{d} \Vert h_g\Vert ^2_{C^{\gamma }(H^+\cap B_{1/2})} \int _{H^+\cap B_r} \delta (x)^{2(\gamma -1)} |\nabla f|dx\\&=C_{d} \Vert h_g\Vert ^2_{C^{\gamma }(H^+\cap B_{1/2})} \int _{{{\,\mathrm{\mathbb {R}}\,}}} \int _{\{ x\in H^+\cap B_r: \delta (x)=t\}} \delta (x)^{2(\gamma -1)} d{{\,\mathrm{\mathcal {H}}\,}}^{d-1}(x) dt\\&=C_{d} \Vert h_g\Vert ^2_{C^{\gamma }(H^+\cap B_{1/2})} \int _{{{\,\mathrm{\mathbb {R}}\,}}} t^{2(\gamma -1)} {{\,\mathrm{\mathcal {H}}\,}}^{d-1}(\{ x\in H^+\cap B_r: \delta (x)=t\}) dt \\&=C_{d} \Vert h_g\Vert ^2_{C^{\gamma }(H^+\cap B_{1/2})} \int _0^r t^{2(\gamma -1)} {{\,\mathrm{\mathcal {H}}\,}}^{d-1}(\{ x\in H^+\cap B_r: \delta (x)=t\}) dt \\&\le C_{d} \Vert h_g\Vert ^2_{C^{\gamma }(H^+\cap B_{1/2})} r^{d-1} \int _0^r t^{2(\gamma -1)} dt \\&= C_{d} \Vert h_g\Vert ^2_{C^{\gamma }(H^+\cap B_{1/2})}r^{d+2(\gamma -1)}. \end{aligned} \end{aligned}$$

The rest of the proof follows exactly as in [9], with the constant only depending on *d*, $\gamma$ and the $C^{1,\alpha }$ norm of *D*, but not on the Hölder norm of *g*. (We remark that *g* here is named $\varphi$ in [17].) We thereby conclude that (2.2) is satisfied since

$$\begin{aligned} \int _{B_r(x_0)} |\nabla u|^2 \le C \Vert h_g\Vert ^2_{C^{\gamma }(H^+ \cap B_{1/2})} r^{d+2(\gamma -1)}, \end{aligned}$$

and thus by Lemma 2.6, the analogous interior regularity estimate, and the bound for $\Vert h_g\Vert _{C^\gamma (H^+\cap B_{1/2})}$, we have that *u* is locally $C^{\gamma }$ Hölder continuous with

$$\begin{aligned} \Vert u\Vert _{C^{\gamma }(B_{1/8}\cap \overline{D})} \le C \left( \Vert u\Vert _{C^{\gamma }( B_1(x_0)\cap \partial D )}+ \Vert u\Vert _{L^\infty (B_1(x_0) \cap D))}+1 \right) , \end{aligned}$$

as we wanted to show. $\square$

As a consequence, we obtain directly Proposition 1.2.

#### Proof of Proposition 1.2

Since *D* is bounded and a $C^{1,\alpha }$ domain, up to a rescaling we can pick $x_1,...,x_N \in \partial D$ such that $\partial D \subset \bigcup _{i=1}^N B_{1/2} (x_i)=:S$ and inside each $B_{1 }(x_i)$ we are in the setting of Proposition 2.7. Thus by Proposition 2.7,

$$\begin{aligned} \begin{aligned} \Vert u\Vert _{C^\gamma (S)}&\le \sum _{i=1}^N \Vert u\Vert _{C^{\gamma }(B_{1/2}(x_i)\cap \overline{D})} \\&\le \sum _{i=1}^N C \left( \Vert u\Vert _{C^{\gamma }( B_1(x_i)\cap \partial D )}+ \Vert u\Vert _{L^\infty (B_1(x_i) \cap D))} +1\right) \\&\le C \left( \Vert g\Vert _{C^{\gamma }(\partial D )}+ \Vert u\Vert _{L^\infty ( D)} +1\right) . \end{aligned} \end{aligned}$$

Since *u* is Lipschitz continuous on $D\backslash S \subset \subset D$ with

$$\begin{aligned} \Vert \nabla u\Vert _{L^\infty (D\backslash S)} \le C \left( 1+ \frac{\Vert u\Vert _{L^\infty (D)}}{{{\,\textrm{dist}\,}}^{d+1}(D\backslash S, \partial D)} \right) , \end{aligned}$$

the result follows. $\square$

#### Remark 2.8

For $\gamma < \frac{1}{2}$, it is not a priori given that the boundary datum *g* is the trace of a function in $H^{1}(D)$. For $\gamma \le \frac{1}{2}$ and $g\in C^{\gamma }(\bar{D}) \cap H^{1}(D)$, the previous proof does not work, since we crucially use the minimality if *u*.

#### Remark 2.9

If $\gamma =1$ (i.e. the datum is Lipschitz), then using the same argument as above we recover the result from [9], as expected,This implies local $\gamma$-Hölder regularity for any $\gamma <1$, but not Lipschitz regularity, in the exact same fashion as for the Laplace equation with Dirichlet boundary condition. It remains open whether this result could be improved to show e.g. $\log$-Lipschitz continuity of the solution,

$$\begin{aligned} |u(x)-u(y)| \le C |\log |x-y||\cdot |x-y | \qquad \forall x,y\in \overline{D}. \end{aligned}$$

## Applications of boundary regularity

### Generic uniqueness of minimizers

Since the functional ${{\,\mathrm{\textit{F}_\Lambda }\,}}$ is not convex, in general, there is no reason to expect uniqueness of minimizers. Already in one dimension it is possible to construct a boundary datum *g* giving two nonidentical minimizers. However, the cases with several minimizers are rare and we expect “almost everywhere" a unique minimizer. We start with a general comparison principle, which can be applied to many different contexts.

#### Lemma 3.1

Let *D* be a bounded open domain of ${{\,\mathrm{\mathbb {R}}\,}}^d$, and let $g,g'\in C(\overline{D})\cap H^1(D)$ with $g' \ge g \ge 0$ in *D* and $g'(z)>g(z)>0$ at some *z* in each connected component of $\partial D \cap \{g>0\}$. Then for corresponding minimizers to (1.2), $u_g$ and $u_{g'}$, we have $u_{g'}\ge u_g$ on $\overline{D}$.

#### Proof

Since on $\{u_g =0\}$ the result holds trivially, consider the open set $\Omega _{u_g} = \{u_g >0\}\cap D$.

Define $\tilde{u}(x) :=\max \left\{ u_{g'}(x), u_g(x)\right\}$. Since by a computation ([17, Lemma 2.5])

$$\begin{aligned} {{\,\mathrm{\textit{F}_\Lambda }\,}}(\max \{u_{g'},u_{g}\},D) + {{\,\mathrm{\textit{F}_\Lambda }\,}}(\min \{u_{g'},u_{g}\},D) = {{\,\mathrm{\textit{F}_\Lambda }\,}}(u_{g'},D) + {{\,\mathrm{\textit{F}_\Lambda }\,}}(u_g,D), \end{aligned}$$

we have that $\tilde{u}$ is also a minimizer with $\Delta \tilde{u} = 0$ in $\Omega _{u_g} \subset \Omega _{\tilde{u}}$. Suppose for contradiction that there exists $x_0 \in \Omega _{u_g}$ such that $u_g(x_0) > u_{g'}(x_0)$, that is $\tilde{u}(x_0)= u_g(x_0)$. Let $\mathcal {C}$ be the connected component of $\Omega _{u_g}$ containing $x_0$.

Set $h(x) :=\tilde{u}(x) -u_g(x) \ge 0$, then on $\mathcal {C}$, $\Delta h=0$ and as its minimum value of 0 is attained at $x_0 \in \mathcal {C}$, by the strong maximum principle $h\equiv 0$ and $\tilde{u} = u_g$ on $\overline{\mathcal {C}}$.

We now show for the sake of contradiction that $\overline{\mathcal {C}}$ contains boundary points where $\tilde{u}> u_g>0$. If $\partial \mathcal {C} \subset \{u_g=0\}$, then by the maximum principle, $u\equiv 0$ in $\mathcal {C}$, contradicting $\mathcal {C} \subset \Omega _{u_g}$. Hence $\partial \mathcal {C} \cap \Omega _{u_g} \ne \varnothing$. Let $x_1 \in \partial \mathcal {C} \cap \Omega _{u_g}$, if $x_1 \in \text {int}(D)$, then by interior Lipschitz continuity $x_1$ cannot be a point in $\partial C$, i.e. $x_1 \in \partial D$ with $g(x_1)>0$. Now the whole component $\partial D \cap \{g>0\}$ containing $x_1$ is contained in $\partial \mathcal {C}$ by continuity from Theorem 1.1. But by assumption we have $z \in \bar{C}$ with

$$\tilde{u}(z) \ge u_{g'}(z) = g'(z) > g(z) = u_{g}(z),$$

a contradiction to the fact that $\tilde{u} = u_g$ on $\mathcal {C}$, finishing the proof. $\square$

We are now able to prove the generic uniqueness for the one-phase problem, using the argument from [13, Proposition 1.2], but for a wider class of boundary data.

#### Proof of Proposition 1.3

During the proof we again use the Lipschitz continuity of minimizers, and we recall that we are taking $\Lambda =1$. By Lemma 3.1, minimizers are ordered with respect to the boundary datum, i.e. $t'> t>0$ implies that $u_{t'} \ge u_{t}$.

Let *t* be such that there are at least two distinct minimizers $u_t^1, u_t^2$. Let $u_t^+ = \max \{u_t^1, u_t^2\}$ and $u_t^- = \min \{u_t^1, u_t^2\}$, then

$$\begin{aligned} \begin{aligned} {{\,\mathrm{\textit{F}_\Lambda }\,}}(u_t^+) + {{\,\mathrm{\textit{F}_\Lambda }\,}}(u_t^-)&= \int _{D \cap \{u_t^1 \ge u_t^2\}} |\nabla u_t^1|^2 + \int _{D \cap \{u_t^1< u_t^2\}} |\nabla u_t^2|^2 \\&\quad + |(\{u_t^1>0\} \cup \{u_t^2>0\}) \cap D| \\&\quad + \int _{D \cap \{u_t^1 \ge u_t^2\}} |\nabla u_t^2|^2 + \int _{D \cap \{u_t^1 < u_t^2\}} |\nabla u_t^1|^2 \\&\quad + |\{u_t^1>0\} \cap \{u_t^2>0\} \cap D| \\&= {{\,\mathrm{\textit{F}_\Lambda }\,}}(u_t^1) + {{\,\mathrm{\textit{F}_\Lambda }\,}}(u_t^2). \end{aligned} \end{aligned}$$

As $u_t^1$ and $u_t^2$ are minimizers, so are $u_t^+$ and $u_t^-$. Now let $x_0$ be a point where $u_t^1$ and $u_t^2$ differ, i.e. without loss of generality $u_t^1(x_0) - u_t^2(x_0) = \varepsilon$. By Lipschitz continuity, there exists $B_r(x_0)$ where $u_t^1(x_0) - u_t^2(x_0) > \varepsilon /2$. Thus for $\rho = \min \{\varepsilon /3, r\}$, there exists a $d+1$ dimensional ball $B_\rho$ such that $B_\rho \subset \text {epi}( u_t^2) \backslash \text {epi} (u_t^1)$.

Repeating the argument for any *t* with non-unique minimizers gives a collection of disjoint (as minimizers are ordered with respect to the boundary datum) balls. Since there can be at most countably many disjoint open balls in ${{\,\mathrm{\mathbb {R}}\,}}^d$, the proof is finished. $\square$

### Generic regularity of the free boundary

We now prove a slightly weaker version of Theorem 1.4, namely, the case where the boundary data is equicontinuous. The main part is already done in [13, Section 4], it remains only to prove [13, Lemma 4.3] for the larger class of boundary data (i.e. equicontinuous, which then implies the result for continuous data, see Lemma 3.3) instead of equi-Lipschitz).

#### Proposition 3.2

Theorem 1.4 holds under the added assumption that the family $g_t$ is equicontinuous.

#### Proof

For simplicity of the exposition, we assume that $D=B_1$. Fix $\tau \in (0,1)$ and let $x_0$ be a free boundary point, $x_0 \in B_{1/2}\cap \partial \Omega _{u_{t_0}}$. for some $t_0 \in [\tau ,1)$. In view of [13, Lemma 4.3], we need to show that there exists $\kappa =\kappa (\tau , d, \omega , M)>0$ (with $\omega$ the common modulus of continuity of $\{g_t\}$ and *M* its uniform $H^1$ bound), such that

$$\begin{aligned} \sup _{B_{\kappa (t-t_0)}(x)} u_{t_0} \le u_t(x) \qquad \text {for } x \in B_{3/4} \text { and } t\in [t_0,1). \end{aligned}$$

By the assumptions on $\{g_t\}$, we have

$$\begin{aligned} g_{t_0} \ge \tau > 0 \qquad \text { on } \partial B_1. \end{aligned}$$

Applying now Theorem 1.1 ($u_{t_0}$ is continuous up to the boundary with modulus of continuity $\bar{\omega }(d, \omega , M)$), gives $\delta =\delta (\tau , d, \omega ,M)<1/16$ such that

$$\begin{aligned} u_{t_0}> 0 \qquad \text {in } U:=B_1 \backslash \overline{B_{1-8 \delta }}. \end{aligned}$$

Using now the comparison lemma, Lemma 3.1, gives $u_t \ge u_{t_0}$ for $t\ge t_0$ and as $U \subset \Omega _{u_{t_0}} \subset \Omega _{u_t}$,

$$\begin{aligned} \Delta (u_t-u_{t_0}) =0 \qquad \text {in }U. \end{aligned}$$

The rest of the proof follows analogously to [13, Lemma 4.3]. $\square$

We set out to remove the assumption of equicontinuity in the previous statement. The rough idea is to partition a family of continuous (not necessarily equicontinuous) functions into countable subfamilies of equicontinuous functions, apply Theorem 3.2 on each subfamily and then show that the size of the set of functions not falling into any equicontinuous subfamily is small. This is due to the separability of continuous functions.

#### Lemma 3.3

Let $d\ge 1$ and $D \subset {{\,\mathrm{\mathbb {R}}\,}}^d$ a bounded Lipschitz domain. Let $\{g_t\}_{t\in (0,1)}$ be a monotone family of continuous functions in $\overline{D}$, i.e.

$$ t \ge s \quad \implies \quad g_t \ge g_s\quad \text {in}\quad \overline{D}.$$

Then, there exists a countable family of disjoint open intervals $\{I_k\}_{k\in \mathbb {N}}$ with $I_k \subset (0, 1)$ such that

- $$(0, 1)\setminus \bigcup _{k\in \mathbb {N}} I_k$$ is countable,
- $g_t$ are locally equicontinuous for $t\in I_k$. That is, for any $K\subset I_k$ compact, the family $\{g_t\}_{t\in K}$ is equicontinuous.

#### Proof

Let $T \subset (0,1)$ be the set where $\{g_t\}_{t\in (0, 1)}$ is locally equicontinuous, i.e.

$$\begin{aligned} T:= \{ t\in (0,1): \quad \exists \eta >0 \text { s.t. } \{g_s\}_{(t-\eta ,t+\eta )} \text { is equicontinuous} \}. \end{aligned}$$

Since for $t_0 \in T$ and $t\in B_{\eta /2}(t_0)$ (where $\eta =\eta (t_0)$),

$$\{g_s\}_{(t-\eta /2,t+\eta /2)} \subset \{g_s\}_{(t_0-\eta ,t_0+\eta )},$$

it follows that *T* is open in $(\{g_s\}_{(0,1)}, \Vert \cdot \Vert _{L^\infty (D)})$. Next, we show by contraposition that for a fixed $t\in T^c$, either

$$\begin{aligned} \exists \varepsilon>0 : \inf _{s>t} \Vert g_t-g_s\Vert _{L^\infty (D)} \ge \varepsilon \quad \text { or } \quad \exists \varepsilon >0 : \inf _{s<t} \Vert g_t-g_s\Vert _{L^\infty (D)} \ge \varepsilon . \end{aligned}$$

Indeed, suppose it is not true true. Since $\{g_s\}_{(0,1)}$ is monotone, we have

$$\begin{aligned} \begin{aligned} \forall \varepsilon>0,\,&\inf _{s>t} \Vert g_t-g_s\Vert _{L^\infty (D)}< \varepsilon \quad \text { and } \quad \forall \varepsilon >0 ,\, \inf _{s<t} \Vert g_t-g_s\Vert _{L^\infty (D)} < \varepsilon \\ \end{aligned} \end{aligned}$$

that is,

$$ \lim _{s\rightarrow t} \Vert g_t-g_s\Vert _{L^\infty (D)} = 0. $$

Let $\varepsilon >0$ and take $\eta , \delta >0$ such that

$$\begin{aligned} \begin{aligned} |s-t|< \eta&\implies \Vert g_t-g_s\Vert _{L^\infty (D)}< \frac{\varepsilon }{3},\\ |x-y|< \delta&\implies |g_t(x)-g_t(y)| < \frac{\varepsilon }{3}. \end{aligned} \end{aligned}$$

By the triangle inequality,

$$\begin{aligned} |g_s(x)-g_s(y)| \le |g_s(x)-g_t(x)| + |g_t(x)-g_t(y)| + |g_t(y)-g_s(y)| \le \varepsilon , \end{aligned}$$

it follows that $\{g_s\}_{(t-\eta ,t+\eta )}$ is equicontinuous, as $\delta$ does not depend on *s*, giving a contradiction.

It remains to show that $T^c$ is countable. We have

$$ T^c = \underbrace{ \{t \in (0,1): \inf _{s>t} \Vert g_t-g_s\Vert _{L^\infty (D)}>0 \}}_{=:T^c_1} \cup \underbrace{\{t \in (0,1): \inf _{s<t} \Vert g_t-g_s\Vert _{L^\infty (D)} >0 \}}_{=:T^c_2}. $$

Let

$$ J_m := \{t \in (0,1): \inf _{s>t} \Vert g_t-g_s\Vert _{L^\infty (D)} \ge \tfrac{1}{m}\}, \quad \text {for}\quad m \in \mathbb {N}.$$

Then, by definition, we have

$$ T_1^c = \bigcup _{m\in \mathbb {N}} J_m. $$

For any $m\in \mathbb {N}$ and $t_1, t_2\in J_{m}$ with $t_2 \ne t_1$ we have $\Vert g_{t_1} - g_{t_2}\Vert _{L^\infty (D)} \ge \tfrac{1}{m} > 0$, and so the family $\{g_t\}_{t\in J_{m}}$ is a family of continuous functions that are pairwise at distance $\tfrac{1}{m}$. However, $C(\bar{D})$ and then also $\{g_t\}_{t \in J_{m}}$ are separable, that is, there exists a countable subset $I \subset J_{m}$ such that $\{g_t\}_{t \in I}$ is dense in $\{g_t\}_{t \in J_{m}}$. Take $g_t \in \{g_t\}_{t \in J_{m}}$ and $t_n \in I \subset J_{m}$ with $\Vert g_{t_n} - g_t\Vert _{L^\infty (D)} \rightarrow 0$. The lower bound on the pairwise distance implies that $t_n = t$ for all *n* large enough, i.e. for any $t\in J_{m}$, $g_t \in \{g_t\}_{t \in I}$, thus $J_m$ is countable. Since *m* was arbitrary and countability is preserved under countable unions, $T^c_1$ is countable and by the same argument $T^c_2$ is countable as well, and so is $T^c$.

Since $T \subset (0,1)$ is open it can be written as a disjoint union of open intervals, that is $(0,1) = T^c \cup T = T^c \cup \bigcup _{k \in \mathbb {N}} I_k$. Now, $K \subset \subset I_k$, $\{(t-\eta _t, t+\eta _t\}_{t \in K}$ admits a finite subcover; and we take as common modulus of continuity its maximum. $\square$

We now combine Proposition 3.2 with Lemma 3.3 to obtain the generic regularity result for a general (not necessarily equicontinuous) family of continuous boundary data, thus proving Theorem 1.4 in its full generality.

#### Proof of Theorem 1.4

We first treat the case $d=d^* +1$. By Lemma 3.3, let $J_0= (0, 1)\setminus \bigcup _{k\in \mathbb {N}} I_k$ and $\{g_s\}_{s}$ locally equicontinuous for $s\in I_k$ for each *k*. By taking a countable compact exhaustion of $I_k$ and applying Theorem 3.2 in each compact, we deduce that, for each *k*, there is $J_k$ countable such that $\text {Sing}(u_t) = \varnothing$ on $I_k \backslash J_k$. The result follows by setting $J:= J_0 \cup \bigcup _{k \in \mathbb {N}} J_k$.

For the case $d\ge d^* +2$, since the dimension estimate $\dim _{{{\,\mathrm{\mathcal {H}}\,}}} \text {Sing}(u_t) \le d-d^*-2$ holds for almost every $t \in K$ and every $K \subset \subset I_k$, it holds a.e. on $I_k$. Hence it holds also almost everywhere on (0, 1). $\square$

## Data Availability

Data sharing is not applicable to this article as no datasets were generated or analysed.

## Footnotes

1 That is, on a sufficiently regular domain *D*, given a boundary datum *g*, the solution *u* “inherits” (in some sense) the regularity of *g*.
